# Investigating contributors to performance evaluations in small groups: Task competence, speaking time, physical expressiveness, and likability

**DOI:** 10.1371/journal.pone.0252980

**Published:** 2021-06-10

**Authors:** Lucie Nikoleizig, Stefan C. Schmukle, Maurin Griebenow, Sascha Krause

**Affiliations:** University of Leipzig, Leipzig, Germany; Public Library of Science, UNITED STATES

## Abstract

This study compared the impacts of actual individual task competence, speaking time and physical expressiveness as indicators of verbal and nonverbal communication behavior, and likability on performance evaluations in a group task. 164 participants who were assigned to 41 groups first solved a problem individually and later solved it as a team. After the group interaction, participants’ performance was evaluated by both their team members and qualified external observers. We found that these performance evaluations were significantly affected not only by task competence but even more by speaking time and nonverbal physical expressiveness. Likability also explained additional variance in performance evaluations. The implications of these findings are discussed for both the people being evaluated and the people doing the evaluating.

## Introduction

Many important interpersonal decisions in everyday life, especially in academic and professional contexts, are made on the basis of impressions of competence or performance evaluations [1, 2]. The positive consequences of being evaluated as high performing have been shown for various aspects of interpersonal interactions: (a) When a speaker (e.g., in politics or television) is evaluated as competent, his/her statements are perceived as more convincing and persuasive by others [3]; (b) groups give influence and power to members who are considered competent [4, 5], for example, by nominating them as leaders [6]; and (c) being evaluated as high performing is a catalyst for career success; for example, employees who are perceived as more competent than other team members get promoted more quickly and more often [7]. As high performance is rewarded with numerous incentives, people strive to be evaluated as high performing and successful in different domains of their professional lives [8].

Because teamwork and collaboration are more and more prevalent in today’s working environments, and interpersonal performance evaluations increasingly take place in these contexts [9, 10], we aim to investigate factors that influence these interpersonal performance judgments in groups. The evaluation of team members’ task performance is determined by their actual task competence but might also be additionally affected by further aspects that are independent of the specific content of the task. According to Driskell and colleagues, observers’ expectations of competence play a particularly important role here [4, 11]. Thus, initial assumptions about a person predispose observers to evaluate a person more (or less) positively. Such initial assumptions are formed by observable cues, that is, the external characteristics (e.g., physical attractiveness, gender, skin color [12]) or the behavior of the evaluated person (e.g., communication behavior [13]). This implies that an individual who wants to be evaluated as high performing (e.g., applicants, employees, students) could additionally focus on changeable aspects and behaviors that are independent of the task’s content in order to evoke the assumption of being a high-performing team member. Vice versa, people (e.g., personnel managers, leaders, teachers) who want to provide accurate evaluations of individual performance in group settings need to be aware of that their performance evaluations might be affected by diverse factors.

**Fig 1:**
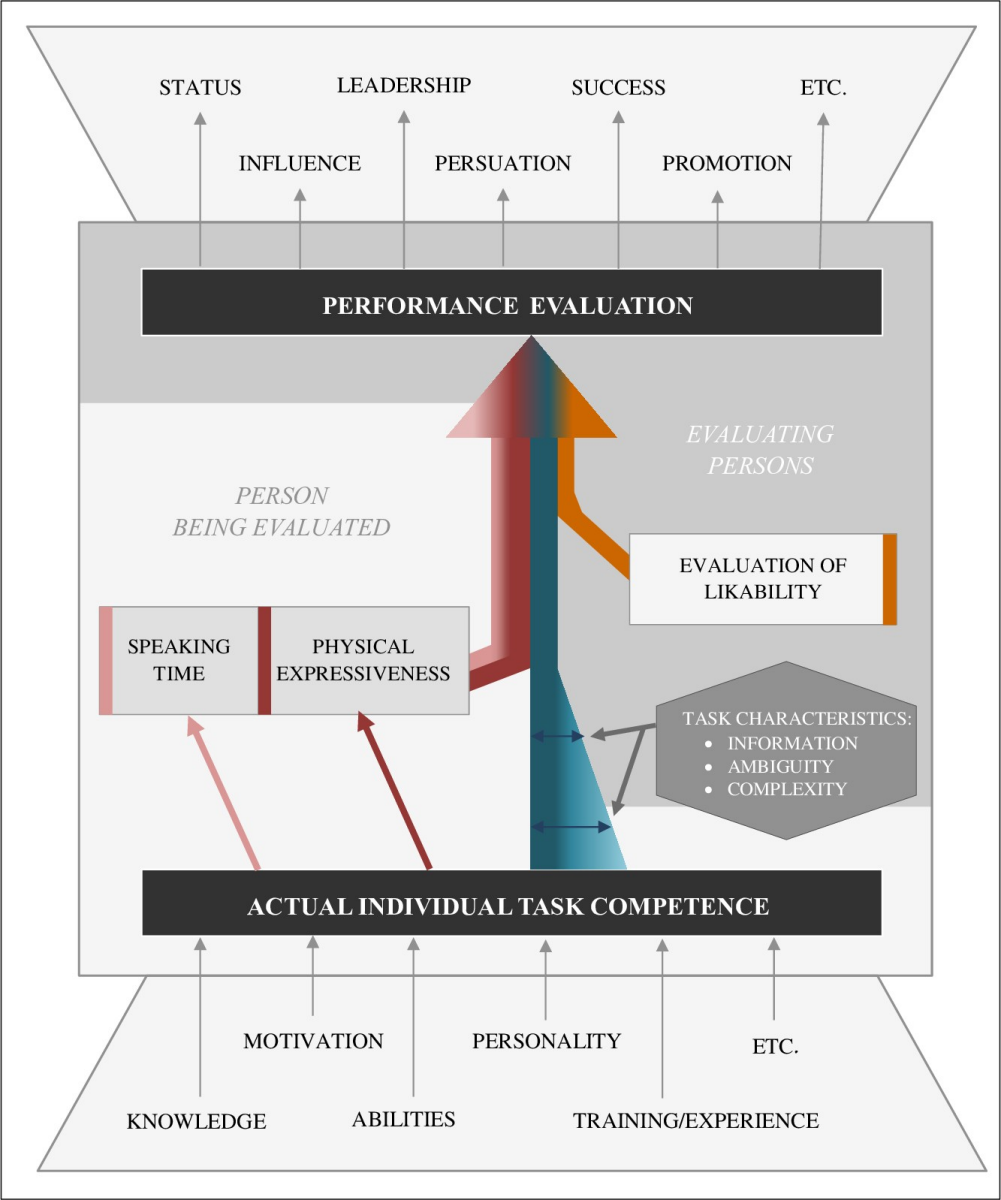
Framework for the performance evaluations in the group tasks.

Thus, in investigating contributors to performance evaluations in the present study we focus on behavioral cues affecting observers’ expectations of competence besides the individuals’ actual task competence. In line with and based on previous research, we developed a framework that illustrates impacts of actual task competence, speaking time and physical expressiveness as aspects of communication behavior, and likability as relevant contributors to performance evaluations made by others in team settings. The components of this framework were derived as follows and are illustrated in Fig 1.

### Task competence and evaluations of performance in teams

People with higher task competence are able to achieve the requirements of a task more successfully [14, 15]. Thus, judgments of individual performance in teams should—at least in part—reflect individual competence, which itself is determined by task knowledge, general cognitive abilities, skills, and motivation [16], personality (especially high conscientiousness [17, 18]), and task experience [19].

However, individual competence might not be the only contributor to performance evaluations, particularly for team tasks in which individual performances cannot be easily observed or attributed to an individual [14, 20]. That is, although some tasks induce situations that provide clear information about the impact of each team member (e.g., additive tasks such as snow shoveling or relay races) and allow team members’ contributions to be deduced easily, in most tasks in current working environments, each team member’s actual performance is less easy to quantify (e.g., in a complex project with high interdependence and discussion between the team members). Here, relevant information on individuals’ actual task competence is less obviously available as the ambiguity and complexity of the task increases [20–22], and additionally other factors might play a role in the evaluation of performance. Thus, in terms of content, a person’s actual competence is a contributor on which an evaluation of the person’s performance should be based, but depending on the availability of information about the person’s actual individual performance, the influence of competence on performance evaluations varies (illustrated by the different degrees of thickness of the blue arrow in Fig 1).

In line with this idea, Sanchez-Cortes, Aran, Mast, and Gatica-Perez measured individuals’ competence in a problem-solving task by asking them to first work on the task individually [23]. Afterwards, participants had to discuss the problem in small groups and find a common solution to the problem-solving task. The group discussion reflected a rather complex situation containing various relevant and irrelevant aspects for evaluating team members’ performances—very similar to situations that occur in everyday work life. Given this complexity, the team members’ competence had only a small impact on participants’ mutual performance evaluations after the group discussions (*r* = .22, *p* = .04). This led to the question of which additional factors influence others’ performance evaluations in addition to the actual competence of the evaluated person in such complex group tasks.

### Aspects of communication behavior and likability as contributors to performance evaluations

In group tasks, team members coordinate their actions primarily through communication behavior, which makes communication a critical behavior for success in group situations. Moreover, communication behavior can be related to actual task competence because it can also be used to convey an individual’s actual level of competence to the other group members. Thus, aspects of communication behavior might be used as omnipresent available sources of information [24] by observers when evaluating a person’s performance in a small group task.

Indeed, previous research showed that team members with a high verbal participation rate are expected to be more competent in group tasks: For example, Sorrentino and Boutillier conducted a study in which small groups worked on a problem-solving task [13]. One team member was a confederate and manipulated the extent to which he verbally participated in the discussion in different groups. The higher the confederate’s amount of speaking time, the higher his team members rated his performance.

Not only verbal but also nonverbal communication increases performance evaluations: Maricchiolo and colleagues presented videos of speakers to participants [3]. Participants gave the speakers higher competence evaluations when the speakers used nonverbal communication behavior (i.e., gestures) while speaking. Thus, speaking for long periods and using expressive gestures might both be behavioral status cues. When an individual communicates a great deal (verbally and nonverbally), observers infer that the individual has a high status; consequently, consistent with the status position, observers evaluate this individual as high performing [4, 11]. In line, both verbal and nonverbal aspects of communication behavior might affect performance evaluations, as illustrated by the pink (speaking time) and red (physical expressiveness) arrows in Fig 1.

Besides communication behavior, likability reflects an additional contributor to performance evaluations in groups (orange arrow in Fig 1). In the present study, likability is defined as one person’s spontaneous positive or negative evaluation of another person. Thus, likability reflects a salient dimension of interpersonal evaluations that affects performance evaluations according to the halo effect [25]. Accordingly, empirical research has provided evidence that the performance evaluation of a person is rated higher when the evaluated person is more likable to the observer [26].

### Present study

In line with our framework (see Fig 1), the present study was designed to explore the various factors that affect people’s evaluations of an individual’s performance in addition to and independent of the individual’s actual level of competence. To do so, we conducted a laboratory study with a design that was parallel to that of Sanchez-Cortes and colleagues [23], in which individuals first completed a task alone to provide a measure of their individual competence in completing the task; afterwards, they had to find a common solution to the problem by negotiating and choosing options together in a group discussion.

For the first time, in the present study, we concurrently examined all components in the framework in Fig 1 in one study. Thus, this study extends previous research by identifying and comparing the relative influences of actual task competence, speaking time, physical expressiveness, and likability on others’ performance evaluations. The interrelatedness of these four contributors to performance evaluations [3, 26–29] and our goal to explain additional influences on performance evaluations besides the impact of actual competence suggest that a hierarchical approach would be optimal for investigating these contributors. In Fig 1, the hierarchical structure of the investigated variables is represented by their staggered contributions to the large overall arrow: In a first step, the impact of actual task competence on the performance evaluations made by others is investigated. Second, speaking time and physical expressiveness as aspects of communication behavior are added to obtain information about the impact of communication behavior on performance evaluations independent of task competence. Finally, likability is added to investigate its impact independent of interindividual differences in task competence, speaking times and physical expressiveness.

One might expect that in addition to the proposed additive effects in our model, the aspects of verbal and nonverbal communication behavior might affect the correlation between task competence and others’ performance evaluations as moderators. Thus, someone who does not speak or move in the group discussion is not able to show his/her competence and cannot be judged validly, whereas someone who communicates and expresses a lot is able to demonstrate his/her (in)competence in the discussion. Further, from a mediation perspective, one could argue that competence affects others’ performance evaluations because it is at least partly conveyed via aspects of communication behavior, that is, task competence leads to longer speaking times and more physical expressiveness (see the pink and red arrows in Fig 1), which in turn affects others’ performance evaluations. To address these mechanisms, the present study additionally investigates the mediating and moderating roles of speaking time and physical expressiveness.

A noteworthy feature of our study is that we used a zero-acquaintance design, i.e. participants did not know each other, have never interacted before the study, and were encountering each other for the first time. Using unacquainted participants to study the contributors to performance evaluations in small groups has the methodological advantage that all interpersonal evaluations cannot be confounded with effects of mutual (prestudy) acquaintances (see, e.g., mere exposure effects on likability [30]). Thus, all investigated groups began their interactions in the first stage of group formation according to Tuckman [31].

Another characteristic of this study is that we investigated our research question in two different performance evaluation settings. First, involved team members who participated in the group discussion evaluated the performances of their team members. They were unaware of the correct solution to the unfamiliar task and they had to evaluate their team members’ performance with limited prior knowledge and without any systematic experience in interpersonal evaluations. Second, qualified observers who observed the discussion from the outside were asked to evaluate the performances of the people engaged in the discussion. The qualified observers were experts on the group task (i.e., they knew the correct solution), they were educated in interpersonal judgments (i.e., bachelor’s degree in psychology and observations training before the study), and they observed and evaluated every participant in our study. On the basis of the differences in quality and amount of available information between these two settings of observing and evaluating others, we were interested in whether group members and qualified observers use the investigated contributors when evaluating performance and how they differ in their weighting of the contributors. Accordingly, we hypothesized that the qualified observers’ performance evaluations should be determined more by the actual competence of the person being evaluated than the team members’ performance evaluations because qualified observers knew the right solution of the task. We therefore assumed that speaking time and physical expressiveness as well as likability may have less influence on qualified observers’ performance evaluations.

## Method

The ethical standards of the American Psychological Association were followed in conducting the research. The ethics committee of the German Psychological Society approved the study procedure. The present study was part of an extensive data set that was collected to answer several independent research questions. Thus, other parts of the data set were previously investigated to examine two different research questions, that is, a) how implicit and explicit liking affect unique friendly behavior (see Study 2 in [32]), and b) how individual performance, interpersonal attraction, and interpersonal behavior affect actual group performance [33]. We provide a complete account of all variables that were assessed in the whole data collection, including a more detailed description of the procedure and additional measures in the S1 File.

### Participants

We gathered data from 164 unacquainted individuals. Participants were German students from two universities (Münster and Leipzig) and different fields of study. Inclusion criteria to participate in the study were German as native language and enrollment as a student. Thus, our sample represents a broader population of German students. Participants had an average age of 24.64 years (SD = 3.44, Min = 20, Max = 38).

Participants were assigned to 41 same-gender groups, each consisting of four members (92 women in 23 all-female groups and 72 men in 18 all-male groups). After the end of the study, each participant received monetary compensation (20 €).

### Procedure

After participants were recruited through announcements, they were asked to register on a website. Here, participants were instructed to book an appointment for a study session that included unacquainted individuals of the same gender. After arriving at the laboratory, participants provided written informed consent to participate in the study. First, they individually solved the National Aeronautics and Space Administration’s (NASA’s) established problem-solving task “Lost on the Moon” [34]. Then, the team was asked to solve the same problem as a team to find a common solution. They were told that they were members of a space crew that had crash-landed on the moon and that they needed to get back to their mothership, which was 200 miles away. The task was to rank a list of 15 items with respect to each item’s importance for the crew’s survival (see S2 File for the exact wording of the task and items of the moon landing task and the correct solution as suggested by NASA). The time limit was 10 min for individuals and 20 min for the group discussion. The time limit was sufficient because all participants were able to completely solve the individual task before the time had run out. All groups also came to an agreed upon ranking in the 20 min that were allotted; group discussions lasted an average of 14 min 51 s (*SD* = 4 min 15 s). During the group task, video cameras recorded the team members’ interactions, which were later analyzed by qualified observers. After the group discussion, participants were seated at computers in separate cubicles to evaluate each team member’s performance and likability.

The moon landing task is a classical discussion task with good properties that allow for evaluations of actual and perceived competence (e.g., [34]). We used the moon landing task because it combines various facets of competence, and it is similar in complexity to typical tasks in real-life work contexts. Creativity, straight reasoning, and knowledge are necessary to solve the task efficiently as an individual. In group settings, the moon landing task offers the opportunity to discuss, negotiate, and choose options several times. Thus, a lot of interpersonal interaction can take place, thus facilitating observable communication behavior.

### Measures

#### Individual task competence

To operationalize participants’ individual task competence, we measured their individual performance, when they solved the moon landing task alone before the group interaction. We compared each item’s rank assigned by each individual with the item’s rank in NASA’s expert solution; absolute differences between these two ranks were calculated. The higher an individual’s summed differences across all the items, the worse the individual’s solution was considered. We recoded the data so that individuals with better performances had higher values; a maximum of 112 points could be achieved for task competence.

#### Speaking time

We operationalized participants’ verbal communication behavior as the amount of time they spent speaking in the group discussion in order to clearly separate it from the nonverbal component of physical expressiveness and the competence-related content of communication behavior. On the basis of the recorded videotapes, each team member’s speaking time was measured with observation software (Observer XT, Noldus Information Technology). We measured only pure speaking times (i.e., breaks in monologues were not taken into account). Also, breaks between speaker changes were not considered. However, lexical productions such as “hmm” or “um” were considered part of a participant’s speaking time. Overlapping speech between conversation partners was assigned to the individuals involved. The total speaking time was computed as the sum of the individual speaking times. To compute participants’ verbal communication behavior, we divided each member’s speaking time by the team’s total speaking time and turned the values into percentages. Note that we focused on speaking times, i.e. on the quantity of each person’s verbal communication behavior independently of the content they communicated.

#### Physical expressiveness

Using the videotapes, six independent observers (five women; all upper-semester undergraduate students in psychology, who conducted the observations as a part of their graduation thesis in their final year) evaluated physical expressiveness with a one-item measure (“How physical expressive is this person?”) on a 6-point Likert scale ranging from 1 (*not at all*) to 6 (*very much*). Observers were given instructions that provided a precise definition of physical expressiveness as an aspect of nonverbal communication behavior: Indicators of strong expressions of physical expressiveness involved movement of the hands and arms, “talking with the hands and feet,” physical active communication, and head movements. Accordingly, an indicator of low expressions of physical expressiveness was a lack of movement (see S3 File for the observation manual). Observer agreement was highly satisfactorily ICC(2, 6) = .88. To ensure that the observers could focus on physical expressiveness and were not distracted by the participants’ spoken words, the videos were presented without sound. Again, we focused on the quantity (vs. the quality) of physical expressiveness to separate this aspect from competence-related aspects of nonverbal communication behavior. Furthermore, the observers were supposed to obtain a comprehensive overview of a participant’s physical expressiveness by forwarding and rewinding the videotapes for a set time of 3 min per participant. This procedure is an established method in behavioral observation designed to ensure that nonverbal behavior is evaluated regardless of the participant’s relative speaking times because each participant is observed for the same length of time [35].

#### Performance evaluations by team members

After the end of the group task, each participant anonymously stated her or his evaluation of each team member’s performance in a round robin design [36]. To evaluate their team members, participants were separated and presented a standardized picture (taken earlier) of each team member’s face on a computer screen, along with three questions: “To what extent did this person make valuable contributions to the group?”; “To what extent was this person effective?”; and “To what extent did this person perform well?” These questions had to be answered on a 4-point Likert scale ranging from 1 (*not at all*) to 4 (*very much*). The internal consistency of this three-item measure was α = .83.

We used the social relations model (SRM; [36]) to analyze the round robin data of team members’ performance evaluations because it allowed us to disentangle different components that are considered in interpersonal judgments. In the SRM, each interpersonal judgment consists of a perceiver effect (e.g., how much a person perceives the other team members as high or low performing on average), a target effect (e.g., how much a person is perceived as high or low performing by the other team members on average), and a relationship effect (e.g., how person *i* perceives the particular performance of person *j*). To address how a person’s performance was perceived by the team members, we used the target effect of the performance ratings as the outcome. We used the R package *TripleR* [37] to extract the target effects of the performance evaluations for each team member.

#### Team members’ perceptions of likability

After the evaluation of performance, each participant was asked to evaluate each team member’s likability. Again, participants were presented a picture of each team member’s face on their computer screen along with one question: “How likeable do you find this person?” The rating was made on a 6-point Likert scale ranging from 1 (*not at all*) to 6 (*very much*). Target effects of likability for each team member were extracted via *TripleR*.

#### Performance evaluations by qualified observers

We presented the videos of the 41 teams (*N* = 164) to three qualified observers (two women; all postgraduate students in psychology) who were blinded to the study hypotheses. The observers watched the videos completely (mean duration about 15 min per team) in a randomized order to prevent observer drifts and sequence effects. The qualified observers answered the same items as the group members, but there were three ways in which the observers were in a better position than the team members to make their performance evaluations. First, the observers were familiar with the moon landing task (i.e., they knew the correct solution for each of the 15 items in the task). Second, the observers were postgraduate students in psychology and had participated in a training session to prepare for the study. Thus, they were educated in assessment and errors in human perception and were aware of the typical ways in which interpersonal evaluations can be biased. In the training session, several example videos of a pre-study were used to familiarize the observers with the observation and evaluation process. The three observers developed a common understanding of their performance evaluations on the basis of the items to be answered by discussing their performance evaluations of the team members in the example videos. They underwent both behavioral observation training and frame of reference training [38]. Third, the observers evaluated all of the 164 participants rather than only three team members, which led to a much more comprehensive overview of performance across participants.

The observers were instructed to watch the videos through the eyes of personnel managers, that is, they were asked to generate evaluations of the participants’ performance on the basis of the participants’ behavior in the discussion. After a video ended, each observer answered the same questions the team members had answered about the participants’ performances on a 6-point Likert scale ranging from 1 (*not at all*) to 6 (*very much*). The internal consistency of this three-item measure was α = .91. The evaluations of each observer and question were aggregated into a mean score for performance evaluations for each participant (observer agreement: ICC [2, 3] = .77). Additionally, we asked the observers to evaluate the extent to which each team member was appropriate for being a leader (ICC [2, 3] = .76). Actually, observers’ performance evaluations and the leadership evaluation were correlated *r* = .93, *p* < .001, which ensures the validity of our performance measure.

#### Qualified observers’ perception of likability

The qualified observers were also asked to rate each participant’s likability (ICC [2,3] = .53) on a 6-point Likert scale. They provided the likability ratings from their own individual perspective by answering the item “How likeable do you find this person?”. The observer’s evaluations of likability were also aggregated into a mean score for each participant.

### Level of data analysis

We had clustered data with participants nested in teams that each consisted of four members. In line with our research question on contributors to performance evaluations at the individual level, we focused on the variability within teams and computed fixed effects regressions [39]. In accordance with Kenny’s suggestions, each participant’s values were adjusted by the means of the participant’s team to address the clustered data structure [36].

Although our research question addressed the individual level of the data, we also looked at differences at the group level, but we found no substantial variance between teams for the performance evaluations of the team members: Differences between teams were even smaller than expected when participants were randomly assigned to the teams, which resulted in a negative ICC = -.19, 95% CI [-.24, -.11]. This effect can be explained by the fact that the team members only had information from their team to make their performance evaluations. Thus, they comprehensively contrasted within their teams and not between teams. For the qualified observers’ evaluations of performance, there was again no significant variance between teams, ICC = -.07, 95% CI [-.15, .07].

## Results

We embrace the values of open science. The data and analysis scripts can be retrieved from https://osf.io/ncjdf/?view_only=865c2f0a0b294a478ab0f36e548436df.

### Descriptive statistics

Table 1 summarizes the descriptive statistics for the performance evaluations (rated either by team members or qualified observers), task competence, speaking time, physical expressiveness, and likability. Intercorrelations are reported for both the original overall data and for within-team variability, where the variables were adjusted by the respective group mean. As can be seen in the first two columns and the first two rows of the correlation matrix in Table 1, team members’ and qualified observers’ performance evaluations were significantly predicted by task competence, speaking time, physical expressiveness, and likability. Note that of all the predictors, the communication behaviors (i.e., speaking times and physical expressiveness) had the highest correlations with performance evaluations.

**Table 1:** Descriptive statistics and intercorrelations.

		Descriptive Statistics		Intercorrelations						
		*M*	*SD*	1	2	3	4	5	6	7
1	Performance (rated by team members) (1–4)	2.93	0.45	-	.70**	.28**	.63**	.47**	.33**	.41**
2	Performance (rated by qualified observers) (1–6)	3.83	0.91	.79**	-	.40**	.70**	.52**	.20*	.59**
3	Task competence (0–112)	68.18	11.17	.34**	.42**	-	.19*	.17*	.22**	.37**
4	Speaking time (in %)	25.00	9.16	.67**	.78**	.22**	-	.47**	.12	.24**
5	Physical expressiveness (1–6)	3.19	0.99	.52**	.58**	.18*	.55**	-	.35**	.48**
6	Likability (rated by team members) (1–6)	4.33	0.66	.30**	.23**	.30**	.14	.31**	-	.35**
7	Likability (rated by qualified observers) (1–6)	3.69	0.76	.42**	.55**	.39**	.30**	.49**	.33**	-

Rating scale values are given in parentheses. Means and standard deviations refer to the overall data. Coefficients above the diagonal represent Pearson correlation coefficients (*r*) for the overall data. Coefficients below the diagonal represent Pearson correlation coefficients (*r*) for the within-group variability.

* *p* < .05.

** *p* < .01.

### Prediction of performance evaluations

**Fig 2:**
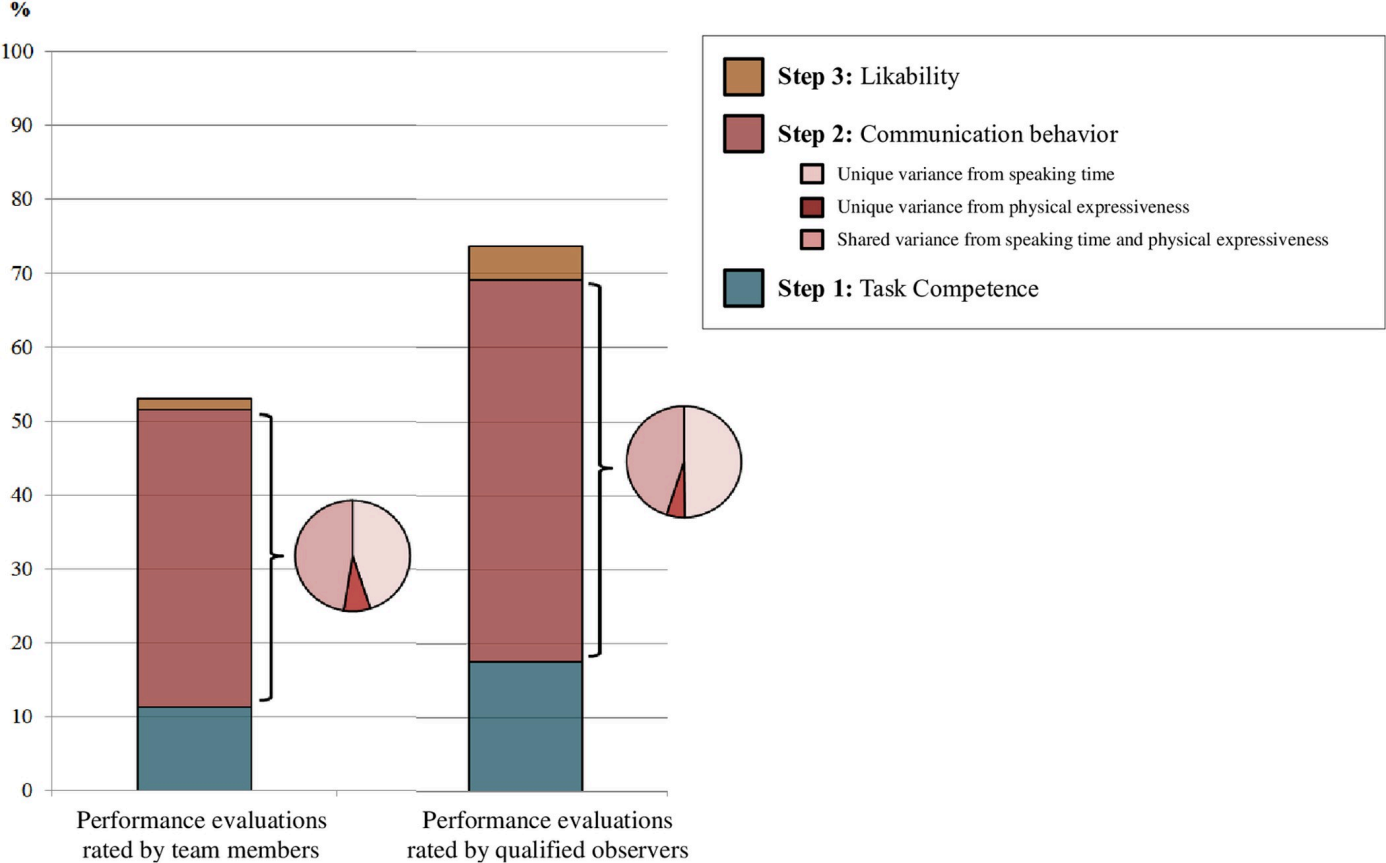
Amounts of variance explained in the hierarchical fixed effects regression for team members’ and qualified observers’ performance evaluations.

In line with our framework, we examined the effects of the variance components of the four contributors to the team members’ and qualified observers’ performance evaluations. To do so, we considered task competence, speaking time, physical expressiveness, and likability as predictors of the performance evaluations in two hierarchical fixed effects regression analyses (see Table 2 and Fig 2).

**Table 2:** Hierarchical fixed effects regression analyses.

	Team members’ performance evaluations						Qualified observers’ performance evaluations					
	*b*	*SE*	*t*	*p*	*R*^*2*^	*ΔR*^*2*^	*b*	*SE*	*t*	*p*	*R*^*2*^	*ΔR*^*2*^
*Step 1*					.113						.176	
Task competence	.015	.004	3.95	< .001			.035	.007	5.10	< .001		
*Step 2*					.515	.402					.692	.516
Task competence	.008	.003	2.83	.005			.021	.004	4.76	< .001		
Speaking time	.024	.004	6.69	< .001			.055	.005	10.00	< .001		
Physical expressiveness	.104	.038	2.72	.007			.187	.058	3.21	.002		
*Step 3*					.530	.015					.737	.045
Task competence	.007	.003	2.24	.027			.141	.004	3.26	.001		
Speaking time	.024	.004	6.90	< .001			.055	.005	10.79	< .001		
Physical expressiveness	.083	.039	2.11	.037			.079	.059	1.34	.181		
Likability ^a^	.098	.051	1.93	.056			.350	.077	4.53	< .001		

^a^ Team members’ perceived likability in predicting team members’ performance evaluations; qualified observers’ perceived likability in predicting qualified observers’ performance evaluations.

Regarding the prediction of team members’ performance evaluations, the results showed that after task competence was accounted for, *R*^2^ = .11, *F*(1, 122) = 15.60, *p* < .001, speaking times and physical expressiveness as aspects of verbal and nonverbal communication behavior together predicted another 40.18% of the variance in the team members’ performance evaluations, *F*(2, 120) = 49.72, *p* < .001. This 40.18% could be divided into 18.09% unique variance from speaking times, *F*(1, 120) = 44.75, *p* < .001; 3.00% unique variance from physical expressiveness, *F*(1, 120) = 7.40, *p* = .008; and 19.09% shared variance from speaking times and physical expressiveness. Additionally, likability predicted another 1.46% of the variance in the team members’ performance evaluations with a marginal level of significance, *F*(1, 119) = 3.71, *p* = .056. Thus, the four predictors together explained 52.98% of the variance in team members’ performance evaluations, *F*(4, 119) = 33.35, *p* < .001.

For qualified observers’ performance evaluations, the results in Table 2 showed that after task competence was accounted for, *R*^2^ = .18, *F*(1, 122) = 26.04, *p* < .001, speaking times and physical expressiveness together predicted another 51.59% of the variance in qualified observers’ performance evaluations, *F*(2, 120) = 100.42, *p* < .001. This 51.59% could be divided into 25.69% unique variance from speaking times, *F*(1, 120) = 100.01, *p* < .001; 2.65% unique variance from physical expressiveness, *F*(1, 120) = 10.29, *p* = .002; and 23.25% shared variance from speaking time and physical expressiveness. Additionally, likability ratings of the qualified observers predicted another 4.53% of the variance in qualified observers’ performance evaluations, *F*(1, 119) = 20.50, *p* < .001. Thus, the predictors together explained 73.71% of the variance in performance evaluations, *F*(4, 119) = 83.40, *p* < .001.

Next, in two different analyses, we compared the impacts of the contributors to performance evaluations by bootstrapping the differences in *R*^2^. The reported confidence intervals were calculated with the adjusted bootstrap percentile (BCa) method.

First, we compared the impact of task competence in predicting the performance evaluations between team members and qualified observers. We found a tendency for task competence to influence performance evaluations rated by qualified observers more than those rated by team members: In two-sided testing, there was no clear evidence of a difference in the impact of task competence predicting team members’ performance evaluations versus qualified observers’ performance evaluations, a difference in *R*^2^ of 0.063, 95% CI [-.001, .129]. When conducting a one-tailed test in accordance with our hypothesis, task competence had a significantly higher impact on qualified observers’ performance evaluations, 90% CI [.011, .119].

Second, when comparing the importance of task competence versus aspects of communication behavior (i.e. speaking time and physical expressiveness) in predicting performance evaluations, we found that for both team members and qualified observers, the aspects of communication behavior were more important than task competence (a difference in *R*^2^ for team members of 0.289, 95% CI [.088, .467]; a difference in *R*^2^ for qualified observers of 0.340, 95% CI [.157, .506]).

Finally, moderation and mediation analyses were conducted to investigate the roles of speaking time and physical expressiveness as aspects of communication behavior in the process of predicting performance evaluations beyond actual task competence. The results of a moderation analysis showed that neither speaking time (*b* = 0.06, *p* = .505) nor physical expressiveness (*b* = -0.09, *p* = .350) moderated the association between task competence and team members’ performance evaluations. Also, when predicting the qualified observers’ performance evaluations, neither speaking time (*b* = 0.03, *p* = .644) nor physical expressiveness (*b* = -0.07, *p* = .290) moderated the association between task competence and qualified observers’ performance evaluations.

**Fig 3:**
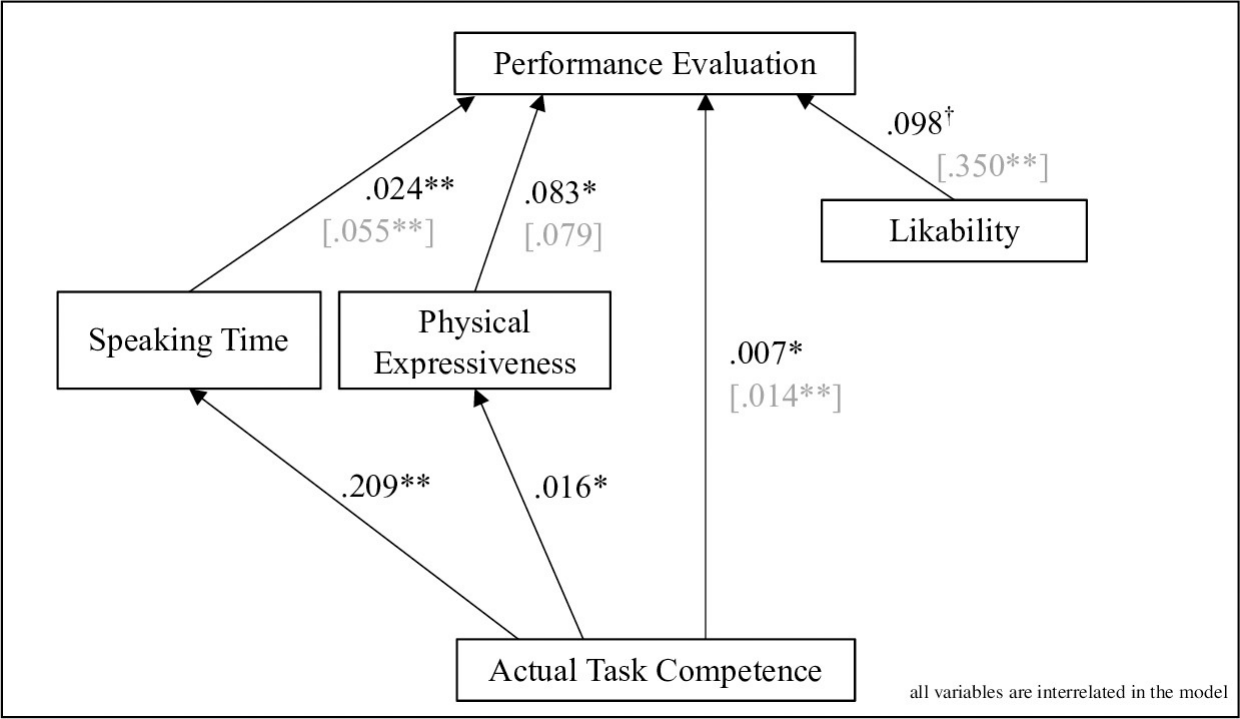
Mediation analysis of performance evaluations made by group members and [qualified observers]; unstandardized path coefficients.

When investigating the indirect effects of competence on performance evaluations via speaking time and physical expressiveness in the mediation analysis, we found a significant partial mediation from speaking time, IE_speakingtime_ = 0.005, *p* = .008, 95% CI [.002, .009], but not from physical expressiveness, IE_expressiveness_ = 0.001, *p* = .103, 95% CI [.000, .004], whereas the direct effect remained significant, DE = 0.007, *p* = .024, 95% CI [.001, .012]. For the unstandardized path coefficients, see Fig 3. These findings indicate that some aspects of an individual’s task competence affect others’ performance evaluations directly, and other aspects are conveyed via verbal communication. In line with the findings of mediating effects on team members’ evaluations, qualified observers’ performance evaluations were also directly affected by competence, DE = 0.014, *p* < .001, 95% CI [.007, .022], and indirectly affected by speaking time, IE_speakingtime_ = 0.011, *p* = .008, 95% CI [.003, .020], whereas physical expressiveness was not a significant mediator, IE_expressiveness_ = 0.001, *p* = .234, 95% CI [.000, .004]. For the unstandardized path coefficients, see Fig 3 again. Further analyses addressing gender differences were not of central interest in our main framework and can therefore be found in the S4 File.

## Discussion

Given the importance of performance evaluations in teams, the present research aimed to provide insight into factors that contribute to the evaluation of individual performances in a typical group task that requires a discussion among team members to find the best solution. We investigated speaking times and physical expressiveness as aspects of verbal and nonverbal communication behavior and likability as contributors that are assumed to influence performance evaluations in addition to the actual task competence of the person being evaluated. Moreover, we compared these contributors with each other and between two different types of observers. The team members’ provided performance evaluations from within the team, whereas qualified observers conducted their evaluations from an external and more qualified perspective.

In line with Sanchez-Cortes and colleagues [23], we found for both team members and qualified observers that actual task competence had a rather small impact on performance evaluations. The actual task competence accounted for only less than one fifth of the variance in performance ratings. This is either because participants did not show their competence in the discussion or because the performance evaluations were influenced by other investigated contributors.

Indeed, we found that aspects of communication behavior affected the others’ performance evaluations independent of the actual competence of the evaluated person. The effect of speaking time was even about four times stronger than the effect of competence and was the most important contributor to performance evaluations. Also physical expressiveness contributed unique aspects to explaining the others’ performance evaluations independently of speaking time. In addition, likability predicted performance evaluations as it was a significant predictor of the qualified observers’ performance evaluations and was almost a significant predictor of the team members’ performance evaluations. These findings are in line with the assumption that performance evaluations are affected not only by actual task competence but also by observable cues (i.e., speaking time, physical expressiveness, and likability) that predispose and therefore bias evaluations of performance [4, 11].

Additionally, we found that speaking times partly mediated the effect from task competence on others’ performance evaluations. That is, task competence led to longer speaking times, which in turn positively affected others’ performance evaluations. Significant moderator effects were not found.

### Implications

Our results suggest that when intending to be evaluated as high performing, simply relying on one’s actual competence might be insufficient, and other relevant aspects in interpersonal interaction might be considered to enhance a person’s performance evaluation. For individuals who want to be evaluated as high performing, it seems to be most important to get a lot of speaking time in group discussions and to additionally include gestures in their communication behavior to give others the impression that they are high performing. Please note that these conclusions apply only to the quantity of speaking time and physical expressiveness that occurred in the sample from the present study. In extreme cases that go beyond normal behavior, excessive talking or domination in small groups could backfire, and excessive physical expressiveness could be perceived as distracting. Thus, too much communication may weaken or reverse the impact on team members’ and qualified observers’ perceptions. Furthermore, making a likable impression is also beneficial for being evaluated as high performing, and this can be accomplished, for example, by smiling, adopting an open body position, exhibiting neat hair and clothes, and engaging in extraverted behaviors [40–42].

For the qualified observers, we expected that performance evaluations would more closely match participants’ actual task competence than the team members’ performance evaluations. The qualified observers had information about the optimal solution of the group task, they were educated in how to make interpersonal judgments, and they watched every participant in the study. They could therefore compare performances in a more differentiated way than the team members could. Indeed, the more highly qualified external observers based their performance evaluations slightly more on task competence than the involved team members. Nevertheless, for the qualified observers, as well, speaking times and physical expressiveness as aspects of competence-independent communication behavior had the greatest impact on their performance evaluations (see [4, 11]). Although we found that the general patterns in the relative proportions of the contributors were quite similar between involved team members and external qualified observers, the investigated contributors explained more variance in the performance evaluations of the qualified observers than of the team members. Thus, indeed, qualified observers based their performance evaluations on actual competence to a greater extent, but they were also more affected by the other competence-independent contributors. Our findings are in line with the literature that has debunked the idea that experts are somehow immune to biases in human processes, even for basic perceptual biases (e.g., in medical settings [43]).

As critical interpersonal decisions are nevertheless made on the basis of performance evaluations in academic and professional life, it is crucial for the people doing the evaluations to conduct valid and thought out performance evaluations. Thus, regarding practical impact, our results could especially contribute to the conceptualization of behavioral observation training sessions in e.g. assessment centers, (that are required by quality assurance initiatives; e.g., ISO 10667, [44]) by supporting suggestions to consider the importance of various contributors in evaluation processes.

### Limitations

In the present study, we investigated some, but not all, of the relevant contributors to performance evaluations. Therefore, further studies in this area should investigate other factors of influence (and their relations to the contributors of the present study) in order to generate comprehensive insights into the prediction of performance evaluations. Further, with our data, we were not able to verify a causal interpretation of our results, and future studies should try to confirm our findings with experimental designs. The sample of our study was representative for unacquainted same-sex groups of German students, but not for the overall German population. Although we do not assume that the contributors influencing performance evaluations differ in other populations, future studies should replicate our findings using nonstudent samples, real work teams and mixed-gender groups. Our findings could also be replicated in other types of group tasks. Perhaps there are group tasks for which individual task competence is more strongly related to others’ performance evaluations than for the moon landing task. These tasks might be more appropriate for drawing inferences from performance evaluations to actual task competence. Nevertheless, it seems natural that performance evaluations in group tasks are generally multi-determined and future studies should focus on investigating the task-specific variability in weighting these contributors in performance evaluations.

## Conclusion

Research on processes and factors that contribute to performance evaluations in teams is highly relevant in professional and academic contexts. With this study, we compared different influences on performance ratings in a problem task that required discussions among team members to find the best solution. We found that task competence had only a rather small impact on performance evaluations and that speaking time and physical expressiveness as indicators of verbal and nonverbal communication behavior were much more important contributors. We observed this pattern of results not only for involved participants who evaluated their other team members but also for external qualified observers who were even aware of the correct solution to the problem. This finding has important implications for applied settings in which measures of individual performances are based on group tasks, such as assessment centers or team projects.

## Supporting information

S1 File Overview and details about the complete study procedure and measures. (PDF)

S2 File Moon landing task. (PDF)

S3 File Behavioral observation manual. (PDF)

S4 File Analysis of gender differences. (PDF)
