# Supplementary material for: Investigating contributors to performance evaluations in small groups: Task competence, speaking time, physical expressiveness, and likability
Source: PLoS One. 2021 Jun 10;16(6):e0252980. doi: 10.1371/journal.pone.0252980 (PMC8191988; doi:10.1371/journal.pone.0252980)
Supplement: S1 File — (PDF) [file pone.0252980.s001.pdf]

Investigating Contributors to Performance Evaluations in Small Groups: Task Competence, Speaking Time, Physical Expressiveness, and Likability

Lucie Nikoleizig, Stefan C. Schmukle, Maurin Griebenow, Sascha Krause

University of Leipzig

Supporting Information

S1: Overview and Details about the Complete Study Procedure and Measures

PLOS ONE

Contact Information

Lucie Nikoleizig: lucie.nikoleizig@uni-leipzig.de

Table of Contents

1. Overview of the Complete Study Procedure and Measures 3

2. Detailed Description of the Study Procedure and Measures 3

3. References S1 7

1. Overview of the Complete Study Procedure and Measures

Participants of the whole project-study underwent the following procedure:

- Questionnaires
- Photographs
- Self-introduction
- Measurement of implicit interpersonal attraction
- Measurement of explicit interpersonal attraction
- Ball-tossing game
- NASA’s “Lost on the Moon” task
- Follow-up survey

2. Detailed Description of the Study Procedure and Measures

Questionnaires. After arriving at the laboratory, participants provided written informed consent to participate in the study. Then, participants were asked to complete various questionnaires. The following list shows the order of the different self-esteem, narcissistic, and personality measures that were completed before the group sessions:

- Rosenberg Self-Esteem Scale (RSES, [1]; German version: [2])
- Adjective scale to assess self-esteem (AS; e.g., [3])
- Narcissistic Personality Inventory (NPI, [4]; German version: [5])
- NEO-Five Factor Inventory (NEO-FFI, [6]; German version: [7])

Photographs. After completing the questionnaires, participants were photographed in a standardized position with a neutral facial expression to create pictures for the implicit and explicit liking measures.

Self-introduction. Next, participants were asked to stand in front of the group and briefly introduce themselves by providing information about their first name, family name, place of origin, field of study, semester, interests, and leisure activities. Afterwards, participants were seated in separate cubicles to complete first an implicit and thereafter an explicit liking measure, both on a personal computer.

Implicit liking measures. We adopted the affective priming paradigm [8] for the assessment of implicit liking in small groups. In the interpersonal attraction affective priming task (IA-APT), participants were asked to identify five pleasant (*favored, honest, cheerful, fair, sincere*) or unpleasant (*arrogant, malicious, mean, annoying, false*) target adjectives as quickly and accurately as possible. In each judgment trial, each target adjective was primed by a picture of a group member's face (500 x 500 pixels) that was previously flashed on the screen. The presentation time of the photos (67 ms) was long enough to identify the group member but too short for a detailed inspection. When an incorrect response was chosen, a red X appeared on the screen for 300 ms. There were two practice blocks and five critical blocks (here, each target word was paired once with each prime; i.e., in the groups of four participants, each test block consisted of 40 trials).

After a filler task that followed the IA-APT, participants completed the response-window affective priming task (RW-APT). In the RW-APT, the same prime and target stimuli as in the IA-APT were used. The time course of the stimuli sequence was also identical, but 300 ms after the target stimulus had appeared, a response window was opened for 150 ms. Participants were instructed to respond to the target stimulus within this time limit. The appearance of a white exclamation point defined the beginning of the response window. If the participant's reaction fell within the window, the white exclamation point turned green. The exclamation point did not change color and disappeared when the participant reacted too slowly. If the participant reacted before the start of the window, the exclamation point never appeared. No feedback was given for incorrect responses. The intertrial interval was set to 1,000 ms. The number of practice blocks and critical blocks was identical to the IA-APT.

Explicit liking measure. After completing the priming tasks, participants were presented a picture of each group member's face, along with four questions: "How *likeable* do you find this person?"; "How *honest* do you find this person?"; "How *sincere* do you find this person?"; and "How *cheerful* do you find this person?" The four adjective ratings to assess

explicit liking were made on a 6-point Likert scale ranging from 1 (*not at all*) to 6 (*very much*). Additionally, participants were asked to rate each group member on two other likeability-independent characteristics: giftedness and beautifulness. All ratings listed up to this point were also assessed as self-perceptions (e.g., "I am likeable") and meta-perceptions (e.g., "This person finds me likable"). After completing the explicit measures, group members interacted with each other in two social settings: First, participants were asked to play an adaptation of the ball-tossing game *Cyberball* [9] on their personal computer. Thereafter, each group had to solve NASA's "Lost on the Moon" problem [10].

Ball-tossing game. Participants were led to believe that they were playing an interactive ball-tossing game with the other group members, although in fact, the other players were simulated by the computer. Before the game began, participants were explicitly asked to visualize the game situation, themselves, and the other player to make their passing behavior more realistic. Furthermore, participants had to hold an 8-digit number in memory while playing the game to elicit more automatically driven reactions [11].

Throughout the game, each participant saw his/her face in the middle bottom field on the screen; the other three players' faces were depicted in the middle left, middle top, and middle right fields on the screen. The game was programmed such that each participant received the same number of passes (40). When the animated ball was tossed to the participant, s/he had to click on one of the other three pictures to pass the ball to the selected group member.

Group task. In NASA's "Lost on the Moon" task, participants were told they were members of a space crew that had crash landed on the moon and needed to get back to the mother ship, which was 200 miles away. The group had to consensually rank a list of 15 items in terms of their importance for the crew's survival (see Supporting Information S2 for the items and the best solution). Before the group tried to solve the moon task together, each group member individually solved the "Lost on the Moon" problem. Afterwards, the group

was asked to solve the moon problem together and to find a common solution. The time limit was 10 min for individuals and 20 min for groups.

Follow-up survey. After the group task, participants were seated at computers in separate cubicles to evaluate each team member's performance and likability. In detail, participants were presented a standardized picture (taken earlier) of each team member's face on a computer screen, along with three questions: "To what extent did this person make valuable contributions to the group?"; "To what extent was this person effective?"; and "To what extent did this person perform well?" These questions had to be answered on a 4-point Likert scale ranging from 1 (*not at all*) to 4 (*very much*). After the evaluation of performance, each participant was asked to evaluate each team member's likability. Again, participants were presented a picture of each team member's face on their computer screen along with one question: "How likeable do you find this person?" The rating was made on a 6-point Likert scale ranging from 1 (*not at all*) to 6 (*very much*). Additionally, participants were asked to rate each group member on two other likeability-independent characteristics: intelligence and beautifulness. All ratings listed up to this point were also assessed as self-perceptions and meta-perceptions.

In the follow-up survey, participants were also asked to give themselves and their group members rankings in terms of performance during the group task. Finally, participants had to answer these items: „ How important was it for you to perform well in this task (e.g., contribute important input)?"; "To what extent were group members able to agree on a common solution?"; "How much did it affect your evaluations that you were also being evaluated by others?"; "How concerned were you about saying the wrong things?"; "How well did you know the moon game before this session today?". Thereafter, participants were debriefed, thanked, and given course credit.

3. References S1

[1] Rosenberg, M. (1965). *Society and the adolescent self-image*. Princeton: University Press.

[2] Collani, G. von & Herzberg, P. Y. (2003). Eine revidierte Fassung der deutschsprachigen Skala zum Selbstwertgefühl von Rosenberg. *Zeitschrift für Differentielle und Diagnostische Psychologie, 24,* 3 – 7.

[3] Brown, J. D., & Kobayashi, C. (2002). Self-enhancement in Japan and America. *Asian Journal of Social Psychology, 5,* 145-168.

[4] Raskin, R. & Terry, H. (1988). A principle components analysis of the Narcissistic Personality Inventory and further evidence of its construct validity. *Journal of Personality and Social Psychology, 54,* 890–902.

[5] Schütz, A., Marcus, B., & Sellin, I. (2004). Die Messung von Narzissmus als Persönlichkeits-konstrukt: Psychometrische Eigenschaften einer Lang- und einer Kurzform des Deutschen NPI [Measuring narcissism as a personality construct: Psychometric properties of a long and a short version of the German Narcissistic Personality Inventory]. *Diagnostica, 50,* 202–218.

[6] Costa, P.T., & McCrae, R.R. (1992). *Revised NEO Five Factor Inventory. Professional manual.* Odessa, FL: Psychological Assessment Resources.

[7] Borkenau, P. & Ostendorf, F. (1993). *NEO-Fünf-Faktoren-Inventar(NEO-FFI) nach Costa und McCrae (Handanweisung)*. Göttingen: Hogrefe.

[8] Fazio, R. H., Jackson, J. R., Dunton, B. C., & Williams, C. J. (1995). Variability in automatic activation as an unobtrusive measure of racial attitudes: A bona fide pipeline? *Journal of Personality and Social Psychology, 69,* 1013-1027.

[9] Williams, K. D., Cheung, C. K. T., & Choi, W. (2000). Cyberostracism: Effects of being ignored over the internet. *Journal of Personality and Social Psychology, 79,* 748-762.

[10] Robins, R. W., & Beer, J. S. (2001). Positive illusions about the self: Short-term benefits and long-term costs. *Journal of Personality and Social Psychology, 80,* 340-352.

[11] Ferreira, M. B., Garcia-Marques, L., Sherman, S. J., & Sherman, J. W. (2006). Automatic and controlled components of judgment and decision making. *Journal of Personality and Social Psychology, 91*, 797-813.
