# Supplementary material for: Investigating contributors to performance evaluations in small groups: Task competence, speaking time, physical expressiveness, and likability
Source: PLoS One. 2021 Jun 10;16(6):e0252980. doi: 10.1371/journal.pone.0252980 (PMC8191988; doi:10.1371/journal.pone.0252980)
Supplement: S2 File — (PDF) [file pone.0252980.s002.pdf]

Investigating Contributors to Performance Evaluations in Small Groups: Task Competence, Speaking Time, Physical Expressiveness, and Likability

Lucie Nikoleizig, Stefan C. Schmukle, Maurin Griebenow, Sascha Krause

University of Leipzig

Supporting Information

S2: Moon Landing Task

PLOS ONE

Contact Information

Lucie Nikoleizig: lucie.nikoleizig@uni-leipzig.de

Table of Contents

1. Individual Task..3

2. Group Task...4

3. Solution Individual and Group Task 5

Please note that the original materials have been translated from German into English. The German versions are available upon request.

1. Individual Task

EMERGENY LANDING ON THE MOON

You are a member of a space crew originally scheduled to rendezvous with a mother ship on the lighted surface of the moon. Due to mechanical difficulties, however, your ship was forced to land at a spot 200 miles from the rendezvous point. The rough landing has ruined your ship and damaged much of the equipment aboard. Only the 15 items listed below were undamaged by the landing. Your survival depends on reaching the mother ship, so the most critical items available must be chosen for the 200-mile trip. The following items are still intact after landing:

	**Your ranking**
• 1 box of matches	
• 1 tin food concentrate	
• 50 feet of nylon rope	
• 30 m^2 parachute silk	
• 1 portable heating unit	
• 2 .45 caliber pistols	
• 1 case dehydrated pet milk	
• 2 hundred-pound tanks of oxygen	
• Stellar map (of the moon's constellation)	
• 1 life raft; self-inflatable with CO2 bottles	
• 1 magnetic compass	
• 5 gallons of water	
• Signal flares (combustible in space)	
• First aid kit containing injection needles	
• Solar-powered FM receiver transmitter	

Your task is to rank the 15 items in terms of their importance for the crew's survival. Indicate your rankings in the table. Put a number 1 by the most important item, a number 2 by the second most important item and so on through number 15, the least important item. Do not give the same ranking to more than 1 item. You have **10-minutes** to complete the rankings.

2. Group Task

EMERGENY LANDING ON THE MOON

You are a member of a space crew originally scheduled to rendezvous with a mother ship on the lighted surface of the moon. Due to mechanical difficulties, however, your ship was forced to land at a spot 200 miles from the rendezvous point. The rough landing has ruined your ship and damaged much of the equipment aboard. Only the 15 items listed below were undamaged by the landing. Your crew's survival depends on reaching the mother ship, so the most critical items available must be chosen for the 200-mile trip. The following items are still intact after landing:

	**Your group ranking**
• 1 box of matches	
• 1 tin food concentrate	
• 50 feet of nylon rope	
• 30 m^2 parachute silk	
• 1 portable heating unit	
• 2 .45 caliber pistols	
• 1 case dehydrated pet milk	
• 2 hundred-pound tanks of oxygen	
• Stellar map (of the moon’s constellation)	
• 1 life raft; self-inflatable with CO2 bottles	
• 1 magnetic compass	
• 5 gallons of water	
• Signal flares (combustible in space)	
• First aid kit containing injection needles	
• Solar-powered FM receiver transmitter	

Your group task is to rank the 15 items in terms of their importance for the crew's survival. Indicate your group consensus of rankings in the table above. Put a number 1 by the most important item, a number 2 by the second most important item and so on through number 15, the least important item. Make sure you argue logically. Only agree to one order if you are convinced that it is correct! Avoid voting. Do not give the same ranking to more than 1 item. You have **20-minutes** to complete the rankings.

3. Solution Individual and Group Task

NASA MODEL SOLUTION

	Position
• 1 box of matches	**15**
• 1 tin food concentrate	**4**
• 50 feet of nylon rope	**6**
• 30 m^2 parachute silk	**8**
• 1 portable heating unit	**13**
• 2 .45 caliber pistols	**11**
• 1 case dehydrated pet milk	**12**
• 2 hundred-pound tanks of oxygen	**1**
• Stellar map (of the moon's constellation)	**3**
• 1 life raft; self-inflatable with CO2 bottles	**9**
• 1 magnetic compass	**14**
• 5 gallons of water	**2**
• Signal flares (combustible in space)	**10**
• First aid kit containing injection needles	**7**
• Solar-powered FM receiver transmitter	**5**
