# Supplementary material for: Investigating contributors to performance evaluations in small groups: Task competence, speaking time, physical expressiveness, and likability
Source: PLoS One. 2021 Jun 10;16(6):e0252980. doi: 10.1371/journal.pone.0252980 (PMC8191988; doi:10.1371/journal.pone.0252980)
Supplement: S3 File — (PDF) [file pone.0252980.s003.pdf]

Investigating Contributors to Performance Evaluations in Small Groups: Task Competence, Speaking Time, Physical Expressiveness, and Likability

Lucie Nikoleizig, Stefan C. Schmukle, Maurin Griebenow, Sascha Krause

University of Leipzig

Supporting Information

S3: Behavioral Observation Manual

PLOS ONE

Contact Information

Lucie Nikoleizig: lucie.nikoleizig@uni-leipzig.de

Please note that the original materials have been translated from German into English. The German versions are available upon request.

Physical Expressiveness - Behavioral Observation Manual

MANUAL

You have to observe the behavior of people in different modalities, situations and under different aspects and to give expert ratings. This task is not easy, on the other hand behavioral observation data are of highest diagnostic and scientific value in psychology.

Please try to concentrate well on your observations, allocate your time well (doing all ratings in one or two weeks leads to low data quality, it is better to observe and evaluate 1-2 hours a day).

In the following manual you will find definitions of the behaviors you should rate (see next page).

Before you start there are some important hints:

1) Each of you has to give your ratings according to the order defined in the rating sheet.

2) That your rating is always related to the right person, please have a look at the picture of the corresponding participant. You should always evaluate the behavior of the "subject".

3) You give your judgements to an individual (it can happen that a video has to be seen several times). Please always pay attention to the first page in the respective rating sheet. Here you will find the study, situation, behavior and modality.

4) With regard to video viewing, the following generally applies:

> The rating sheets are to be edited in a **fixed order** in order to keep the effects of judgement as low as possible.
> Please rate each behavior to the best of your knowledge and belief.
> If you want to assess a person's behavior in the group study, watch the video for **at least 3 minutes**.
> You don't have to watch the video in full length, you are welcome to fast forward or rewind. Get an **impression of the behavior in the video as complete as possible** (i.e. watch the beginning, middle and end).
> If you notice anything or you can't give a rating, please write it down on an extra piece of paper.
> On the first page of your rating sheet you will find the most important conditions of your rating.

Now have fun with the ratings. Please contact me directly if there are any major inconsistencies.

behavior	Indices
**Physical expressiveness**	**high expression:**<br>- gesticulating, "talking with hands and feet".<br>- also head movements such as nod or negate<br>- distinct facial acrobatics (facial expressions) while talking<br><br>**low expression:**<br>- low-movement communication/ no use of the body when acting
