# Supplementary material for: Investigating contributors to performance evaluations in small groups: Task competence, speaking time, physical expressiveness, and likability
Source: PLoS One. 2021 Jun 10;16(6):e0252980. doi: 10.1371/journal.pone.0252980 (PMC8191988; doi:10.1371/journal.pone.0252980)
Supplement: S4 File — (PDF) [file pone.0252980.s004.pdf]

Investigating Contributors to Performance Evaluations in Small Groups: Task Competence, Speaking Time, Physical Expressiveness, and Likability

Lucie Nikoleizig, Stefan C. Schmukle, Maurin Griebenow, Sascha Krause

University of Leipzig

Supporting Information

S4: Analysis of Gender Differences

PLOS ONE

Contact Information

Lucie Nikoleizig: lucie.nikoleizig@uni-leipzig.de

Analysis of Gender Differences

To investigate differences between the all-female teams (i.e., 92 women in 23 groups) and the all-male teams (i.e., 72 men in 18 groups), we report results of hierarchical fixed effects regressions separately for women and men in Tables T1 and T2. Furthermore, we compared the complete regression models (i.e., Step 3 of the hierarchical regression) between women and men. Therefore, we computed a regression model for the all-female groups and the all-male groups without any parameter constraints in a structural equation model. The fit of this model (which was perfect given that the model's degrees of freedom equaled 0) was compared with the fit of a second model in which all path coefficients were set to the same value across the two genders. The results for the second model showed that the model fit the data very well, $\chi^2 = 2.05$, $df = 4$, $p = .73$. Altogether, these results show that there were no significant differences in the parameter estimates between genders.

Table T1

Women: Hierarchical Fixed Effects Regression Analysis

	Team members' performance evaluations						Qualified observers' performance evaluations					
	b	*SE*	*t*	*p*	R^2	ΔR^2	*b*	*SE*	*t*	*p*	R^2	ΔR^2
Step 1					.206						.211	
Task competence	.020	.005	4.20	< .001			.040	.009	4.27	< .001		
Step 2					.476	.270					.640	.429
Task competence	.011	.004	2.75	.008			.019	.007	2.73	.008		
Speaking time	.022	.006	3.76	< .001			.061	.009	6.50	< .001		
Physical expressiveness	.114	.055	2.08	.042			.187	.089	2.11	.039		
Step 3					.491	.015					.724	.084
Task competence	.010	.004	2.30	.025			.100	.006	1.60	.114		
Speaking time	.024	.006	3.99	<.001			.067	.008	7.92	< .001		
Physical expressiveness	.089	.058	1.54	.127			.076	.083	0.91	.364		
Likability [a]	.089	.066	1.36	.179			.486	.110	4.42	< .001		

[a] Female team members' perceived likability in predicting female team members' performance evaluations; qualified observers' perceived likability in predicting qualified observers' performance evaluations.

Table T2

Men: Hierarchical Fixed Effects Regression Analysis

	Team members' performance evaluations						Qualified observers' performance evaluations					
	b	*SE*	*t*	*p*	R^2	ΔR^2	*b*	*SE*	*t*	*p*	R^2	ΔR^2
Step 1					.042						.139	
Task competence	.009	.006	1.53	.133			.030	.010	2.92	.005		
Step 2					.572	.530					.764	.625
Task competence	.005	.004	1.17	.249			.022	.006	3.88	< .001		
Speaking time	.025	.005	5.40	< .001			.051	.006	7.96	< .001		
Physical expressiveness	.094	.053	1.76	.084			.183	.074	2.48	.017		
Step 3					.586	.014					.790	.026
Task competence	.003	.004	0.70	.489			.015	.006	2.44	.018		
Speaking time	.024	.005	5.32	<.001			.049	.006	8.05	< .001		
Physical expressiveness	.077	.054	1.42	.163			.072	.083	0.87	.389		
Likability [a]	.114	.087	1.31	.195			.287	.114	2.53	.015		

[a] Male team members' perceived likability in predicting male team members' performance evaluations; qualified observers' perceived likability in predicting qualified observers' performance evaluations.
